# Application of Ultrafiltration for Recovery of Polyphenols from Rose Petal Byproduct

**DOI:** 10.3390/membranes13100818

**Published:** 2023-09-28

**Authors:** Mariya Dushkova, Alexios Vardakas, Vasil Shikov, Kiril Mihalev, Margarita Terzyiska

**Affiliations:** 1 Department of Process Engineering, University of Food Technologies, 26 Maritza Blvd., 4002 Plovdiv, Bulgaria; 2 Department of Agricultural Technology, Technological Educational Institution of Western Macedonia, Kila, 50 100 Kozani, Greece; alexiosv@gaea.gr; 3 Department of Food Preservation and Refrigeration Technology, University of Food Technologies, 26 Maritza Blvd., 4002 Plovdiv, Bulgaria; vshikov@uft-plovdiv.bg (V.S.); kmihalev@uft-plovdiv.bg (K.M.); 4 Department of Mathematics, Physics and Information Technologies, University of Food Technologies, 26 Maritza Blvd., 4002 Plovdiv, Bulgaria; mterziyska@uft-plovdiv.bg

**Keywords:** ultrafiltration, rose petal byproduct, polyphenols, green technology

## Abstract

One main objective of this study was to increase the utilization of raw material in the rose (*Rosa damascena* Mill.) essential oil industry by the application of membrane technologies. In this research, distilled (dearomatized) rose petals, the primary byproduct in essential oil production, were subjected to an enzyme-assisted extraction and subsequent membrane separation for partial concentration at different levels using UF1-PAN and UF10-PAN membranes. The results show that the permeate flux decreased with a rise in volume reduction ratio and increased with a rise in transmembrane pressure and feed flow rate. At the beginning of the process, the highest flux was with the UF1-PAN membrane, but at the end of the process, it was with the UF10-PAN membrane. Total polyphenols of the retentates increased by 27–39% and 26–67% during ultrafiltration with the UF1-PAN and UF10-PAN membranes, respectively, with the highest value obtained for the UF10-PAN membrane at VRR 6. The highest concentration factor and rejection of total solids, total polyphenols, redox-active antioxidants, and radical scavenging antioxidants were obtained at VRR 6 with the UF10-PAN membrane. The use of green technology based on enzyme-assisted extraction and ultrafiltration for recovery and concentration of polyphenols from rose petal byproduct solves practical environmental problems for the treatment and utilization of byproducts from the rose oil industry. The retentate obtained could be used in the food production, cosmetic, and pharmaceutical industries.

## 1. Introduction

Currently, the replacement of synthetic food additives with their natural equivalents greatly helps satisfy the demands of the food industry due to the trend of healthy human nutrition and rising customer desire for natural foodstuffs.

Foods are a rich source of phenolic acids. Caffeic acid and, to a lesser extent, ferulic acid are the most well-known [1]. Flavonoids are the most populous polyphenols in the human diet. Their appearance is limited to a few foods. The primary source of isoflavones is soybean, which has a genistein and daidzein content ~1 mg/kg [2]. Due to their estrogenic characteristics and potential roles in the prevention of breast cancer and osteoporosis, these two isoflavones have drawn a lot of interest [3]. The primary dietary source for flavanones is citrus fruits. Hesperidin from oranges (125–250 mg/L juice) is the most consumed [4].

The antioxidant activity of *Rosa damascena* extracts and products is mostly reasoned by this high content of phenolic compounds. Among several analyzed rose teas, those prepared from cvs San Francisco (of the hybrid tea roses group), Katharina Zeimet (polyantha group), Mercedes (floribunda group), and from the essential oil rose *R. damascena* exhibited the highest antioxidant activities, which were even higher than those of green tea [5]. Distilled (dearomatized) rose (*Rosa damascena* Mill.) petals, a byproduct from the essential oil industry, are rich source of polyphenols, particularly flavonols [6].

The wastewater from fruit and vegetable processing, as well as byproducts from essential and plant oil production, could create serious environmental problems due to the availability of many important bioactive substances. The food sector has a lot of potential with bioactive substances to improve their process, recover these compounds, and create value-added products, while minimizing environmental effects [7]. One of the traditional methods for recovery is extraction. Factors impacting recovery are important in order to optimize the process and to achieve efficiency, and they are as follows: solvent usage, substrate type, concentration, particle size, temperature, and quality and storage of extracts, as well as the stability of extraction [8].

Compared with traditional separation and concentration methods, membrane processes are low-cost and nonthermal without phase change or chemical agents, as well as having improved product quality and a simple process design [9]. In recent years, membrane methods have garnered important interest for the separation and concentration of bioactive components from plant extracts and byproducts of agro-food industries [10,11,12,13,14,15,16,17,18]. No literature was found on the use of membrane techniques, including ultrafiltration, for the recovery of polyphenols from distilled (dearomatized) rose petals form *Rosa damascena* Mill., which was the aim of this investigation.

## 2. Materials and Methods

### 2.1. Materials

#### 2.1.1. Chemicals

The reagents for analysis were TPTZ [2,4,6-tripyridyl-s-triazine] and gallic acid monohydrate (Fluka, Buchs, Switzerland); Folin–Ciocalteau’s reagent (Merck, Darmstadt, Germany); DPPH [2,2-diphenyl-1-picrylhydrazyl] and Trolox [(+/−)-6-hydroxy-2,5,7,8-tetramethyl-chroman-2-carboxylic acid] (Sigma-Aldrich, Steinheim, Germany). The remaining chemicals were all of analytical grade.

#### 2.1.2. Enzyme Preparations

Commercial enzyme preparations of the following types were used: hemicellulolytic Xylanase AN (Biovet JSC, Peshtera, Bulgaria); cellulolytic Rohament CL (AB Enzymes GmbH, Darmstadt, Germany; pectinolytic Pectinex Ultra Color (Novozymes A/S, Bagsvaerd, Denmark).

#### 2.1.3. Plant Materials

Plant byproduct, a residue from water–steam distillation of oil-bearing rose (*Rosa damascena* Mill.) petals, was supplied by Ecomaat Ltd. (Mirkovo, Bulgaria) during the 2017 processing campaign. After pressing the wet material in a rack and cloth press, the pomace obtained was stored frozen until used. After defrosting, the pomace was hot-air-dried (60 °C, 8 h).

### 2.2. Enzyme-Assisted Extraction of Rose Petal Byproduct

Dry pomace that was finely ground (particle size < 0.63 mm) was mixed with water (12:1, *v*/*w*), acidified (pH 4.0) with 50% (*w*/*v*) citric acid, and then kept overnight at 10 °C for rehydration. The suspension was adjusted to pH 4.0, heated in a water bath at 50 °C for 20 min, and then 10 mL of an acidified water solution (1.2%, *v*/*v*) of the enzyme mixture (1:1:1) was added. The sample was pressed using a rack and cloth press (25 MPa) after 2.5 h of incubation at 50 °C. The extract was filtered through a paper filter after being pasteurized at 80 °C for 4 min.

### 2.3. Methods

#### 2.3.1. Ultrafiltration of Rose Petal Extract

##### Membranes

Polyacrylonitrile (PAN) membranes UF1-PAN and UF10-PAN with a nominal molecular weight cut-off of 1 and 10 kDa, respectively, were used for membrane experiments. The membranes were produced at the University of Burgas, Bulgaria. They were already assembled in a plate-and-frame module but were used for the first time for rose petal extract.

##### Experimental System

**Figure 1:**
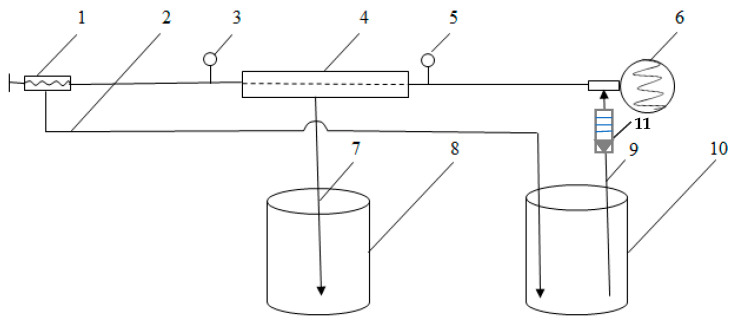
Scheme of laboratory membrane equipment: 1—pressure regulator; 2—pipeline for retentate; 3—manometer (0–1 MPa); 4—replaceable plate and frame membrane module; 5—manometer (0–1 MPa); 6—3-frame piston pump; 7—pipeline for permeate; 8—tank for permeate; 9—pipeline for feed solution/retentate; 10—tank for feed solution/retentate; 11—rotameter.

A laboratory system equipped with a replaceable plate and frame membrane module with an open-flow channel and active membrane area of the module of 0.125 m^2^ was used for the ultrafiltration experiments (Figure 1) [19]. The cross-section area of the feed channel was 0.000071 m^2^. The process was batch mode operation. The concentration determinations were taken during the process.

For each ultrafiltration experiment, 6 L of the feed solution (*V*_F_) was used. The following operating conditions during ultrafiltration were used: transmembrane pressure—from 0.2 to 0.4 MPa, volume reduction ratio (VRR)—from 2 to 6, and feed flow rate—from 190 to 330 dm^3^/h. The feed flow rate was changed and measured by rotameter placed before the pump inlet. The flow rates used in the research were considered as follows: when a flow rate below 190 dm^3^/h was used, foaming occurred, which impacted the pump’s work. A flow rate of 330 dm^3^/h is the maximum one possible for the pump. The working temperature during ultrafiltration was 20 °C. The experimental design was performed as follows: after starting ultrafiltration with a new membrane and when VRR 2 was reached, the flow rate was reduced to 190 dm^3^/h using a rotameter, and a transmembrane pressure of 0.2 MPa was maintained during three consecutive measurements of permeate flux at these conditions. Maintaining the abovementioned flow rate, the pressure was increased to 0.3 MPa using the pressure regulator, and three sequential measurements of flux were performed. The same was performed for 0.4 MPa. Then, the flow rate was increased to 330 dm^3^/h and the pressure was maintained at 0.2 MPa for three measurements of permeate flux, as well as at 0.3 MPa and 0.4 MPa. Then, the concentration of the rose petal byproduct continued until VRR 4 and VRR 6, where the same procedure was repeated. The error was calculated on the basis of repetition of the measurements with a new membrane and not on the basis of repetition of the experiment. To calculate the errors for the concentration factor and rejection, three samples were taken sequentially from the same experiment at one set parameter. After ultrafiltration, the membranes were cleaned with 0.5% NaOH at a temperature of 50 °C, pressure of 0.2 MPa, 30 min circulation time, and final rinsing with distilled water.

After experimental measurements of the permeate’s volume (*V*, cm^3^) separated from the membrane module for the time defined (*t*, s) under different working conditions, the flux (*J*, dm^3^/(m^2^.h)) was calculated using the following formula:

(1) $$J=\frac{V}{A\times t}$$

where *A* = 0.125 m^2^ is the membrane surface area in the module [9].

Rejection (*R*, %) and concentration factor (*CF*) were calculated using the following formulas:

(2) $$R=\left(1-\frac{C_{p}}{C_{R}}\right)\times 100,\;\%$$

(3) $$CF=\frac{C_{R}}{C_{F}}$$

where *C_P_*, *C_R_*, and *C_F_* are the contents of the corresponding compounds in the permeate, retentate, and feed solution, respectively.

The VRR was determined as follows:

(4) $$VRR=\frac{V_{F}}{V_{R}}$$

where *V_F_* is the feed solution’s volume, dm^3^; *V_R_* is the retentate’s volume, dm^3^.

The yield of polyphenols in the end-product was calculated as follows:

(5) $$yield=1-{\left(1-\frac{V_{p}}{V_{F}}\right)}^{\frac{C_{p}}{C_{F}}}$$

where *V_F_* is the feed solution’s volume, dm^3^; *V_p_* is the permeate’s volume, dm^3^; and *C_P_* and *C_F_* are the polyphenolic contents in the permeate and feed solution, respectively.

#### 2.3.2. Flow Diagram

**Figure 2:**
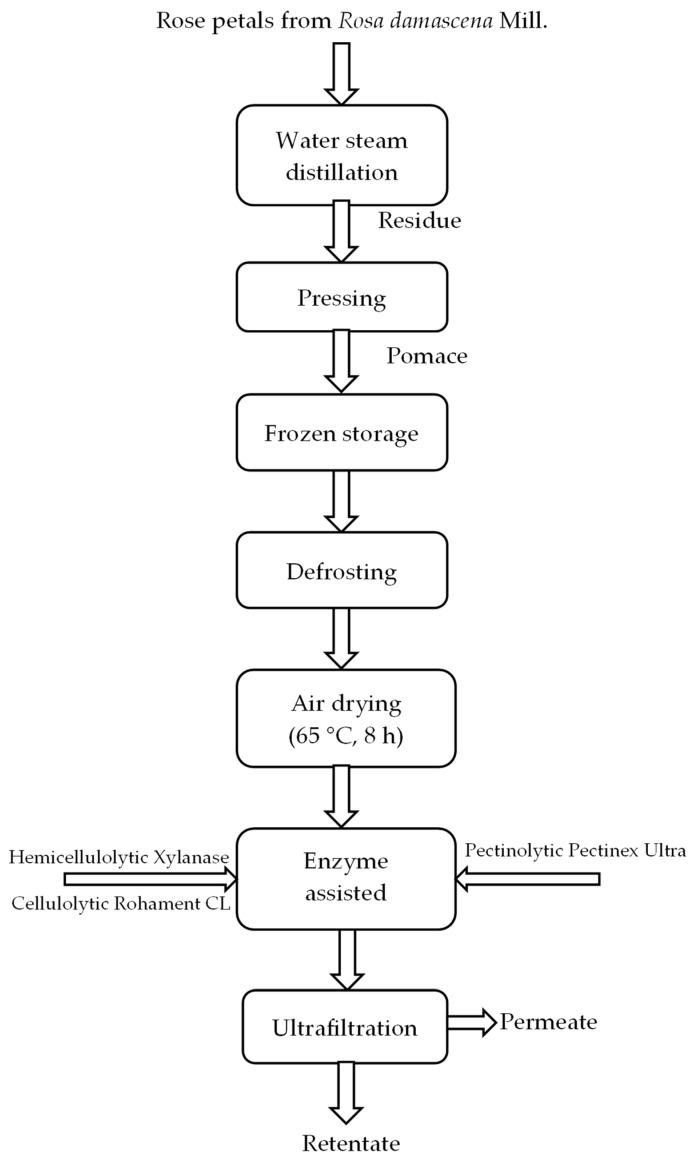
Flow diagram.

The flow diagram of processes and methods is presented in Figure 2.

#### 2.3.3. Phytochemical Analyses

A spectrophotometer Helios Omega UV–Vis fitted with software VISIONlite version 2.1 (Thermo Fisher Scientific, Madison, WI, USA) with 1 cm path length cuvettes was used for all measurements.

##### Polyphenolic Content

The total polyphenolic content (TPP) was established by the method of Singleton and Rossi [20], with modifications described in our previous work [19].

##### Total Antioxidant Capacity

The total antioxidant capacity was determined by the DPPH (free radical scavenging activity) and FRAP (ferric reducing antioxidant power) assay described in our previous work [19].

The method of Brand–Williams et al. [21] with modifications described in [19] was used for DPPH assay.

The method of Benzie and Strain [22] with some modifications described in [19] was applied for FRAP assay.

##### Protein Content

The method of Bradford [23] was used for protein content, and the results are given as mg bovine serum albumin equivalents (BSAE) per 100 g on a dry-weight basis.

#### 2.3.4. Determination of Dry Matter Content

The content of dry matter (total solids) was determined using an MLB 50-3 moisture analyzer (KERN & SOHN GmbH, Balingen, Germany).

#### 2.3.5. Statistical Analysis

The results of the current investigation are given as the averages of at least three determinations. One-way ANOVA in Microsoft Excel 2010 was used to compare the averages utilizing Tukey’s test at a significance level (*p*-value) of 0.05.

## 3. Results and Discussion

### 3.1. Permeate Flux

The pure water flux measured before the experiment was as follows: 164.5 ± 0.7 L/(m^2^.h) for UF1-PAN and 173 ± 0.6 L/(m^2^.h) for UF10-PAN. After ultrafiltration and cleaning of the membranes, the pure water fluxes were 131.7 ± 0.4 L/(m^2^.h) for UF1-PAN and 133.5 ± 0.5 L/(m^2^.h) for UF10-PAN, which demonstrated that fouling of the membrane occured.

**Figure 3:**
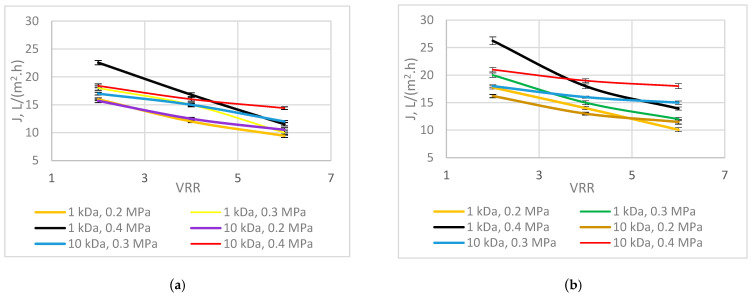
Permeate flux at different process conditions: (**a**) feed flow rate 190 dm^3^/h; (**b**) feed flow rate 330 dm^3^/h.

Figure 3 shows the permeate flux for both investigated membranes at different working conditions. The lowest results for the permeate flux were obtained at *p* = 0.2 MPa, VRR = 6, and Q = 190 dm^3^/h, while the highest wereat *p* = 0.4 MPa, VRR = 2, and Q = 330 dm^3^/h for both membranes. Comparing the flux with the two investigated membranes, it can be seen that at the beginning of the process, the highest flux was obtained with the UF1-PAN membrane, but at the end of the process, the highest flux was obtained with the UF10-PAN membrane (*p* < 0.05). This is probably due to a more pronounced effect of the concentration polarization on the UF1-PAN membrane than on the UF10-PAN membrane. It is also noteworthy that the data for flux collected during the experiment were more influenced by foulilng than those collected at the beginning of the process (with a more “fresh” membrane). Qaid et al., in 2017 [24], found a positive correlation between the permeate flux and molecular weight cut-off of the membrane during ultrafiltration of Valencia orange juice. Acosta et al., in 2014 [25], established that the permeate flux depended on the MWCO of the membrane during ultrafiltration of blackberry (*Rubus adenotrichus* Schltdl.) juice. The same authors found that the effect of pressure on resistance appears to be complex and distinctive, depending on the membrane’s material and its nominal MWCO. It also can be seen from the figure that when the VRR increased, the permeate flux decreased. This is due to the concentration rise in the solutesm which increases dynamic viscosity [26] and leads to a decrease in flux. The same authors established a linear dependence between the permeate flux and transmembrane pressure during the ultrafiltration of pomegranate juice. As reported by Cai et al. in 2020 [27], this phenomenon was related to the cake layer, which increased during the process, and thus, the flux decreased. Similar results were obtained from Gaglianò et al. [28] for diafiltered apple juice, who established that the decrease in the permeate flux differed according to the membrane type. The transmembrane pressure had a positive effect on the permeate flux for both membranes investigated. An explanation for this consists in the fact that a rise in the recirculation velocity benefits the hydrodynamical conditions, decreases the effect of the concentration polarization, and improves the mass transfer coefficient, and thus the flux increases [24]. The feed flow rate led to an increase in the permeate flux. It is also well known that increasing the feed flow improves the mass transfer at the membrane, resulting in higher fluxes. According to Lai et al. in 2021 [29], the cross-flow velocity rise led to a decrease in the concentration polarization on the membrane surface and an increase in the permeate flux. The same authors also found that the permeate flux rise differed according to the membrane’s material. The increased transmembrane pressure can be applied when the flux decreases due to VRR rise.

### 3.2. Phytochemical Characteristics

Total polyphenols of the retentates increased by 27–39% and 26–67% during ultrafiltration with 1 kDa and 10 kDa membranes, respectively, depending on the volume reduction ratio (Table 1). The highest concentration was observed for 10 kDa membrane at VRR 6. However, no significant differences were observed between all VRRs for 1 kDa membrane and between VRR 2 and VRR 4 for 10 kDa membrane (*p* > 0.05). Moreover, the content of total polyphenols was similar for both membranes at VRR 2. Therefore, the concentration process does not simply depend on the molecular weight, i.e., chemical structure, of the rose petal polyphenols, which are mainly flavonols [6].

**Figure 4:**
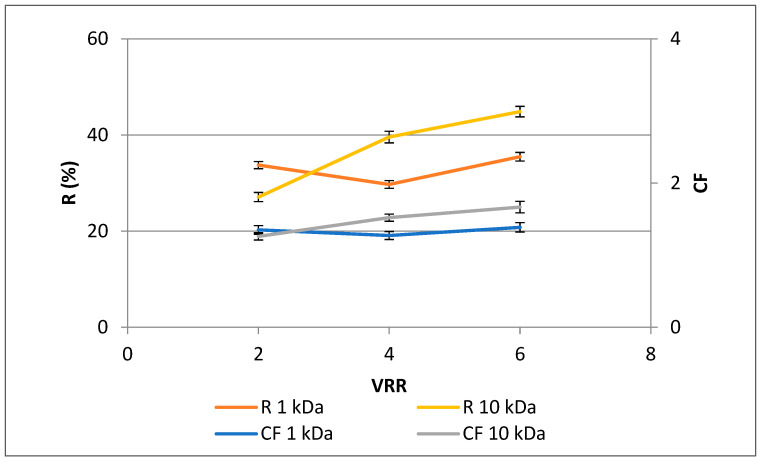
Dependences of concentration factor (CF) and rejection (R, %) of total polyphenols in rose petal extracts on the VRR during ultrafiltration with 1 kDa (UF1-PAN) and 10 kDA (UF10-PAN) membranes.

Plant materials contain polyphenols that may be associated with proteins, cocolored with other substances in the system, or condensed by oxidation. The presence of polyphenols may have a direct or indirect impact on additional ingredients [26]. As shown in Figure 4, higher rejections (*p* < 0.05) were observed for the UF10-PAN membrane than for UF1-PAN for VRR 4 and 6, except VRR 2. The same trend was observed for the concentration factor. An increase in the values of both rejection and concentration factor was established with the concentration level’s rise (VRR) during the ultrafiltration process, except for the concentration factor at VRR 4 for the UF1-PAN membrane. The highest values for the rejection and concentration factor were observed at VRR 6 for UF10-PAN—44.9% and 1.7, respectively. This shows the possibility of ultrafiltration to concentrate the polyphenols. The higher rejection for membranes with larger nominal MWCO could also be explained by the fact that the influence and extent of membrane fouling was different. At the same time, the concentration factors for both membranes at VRR 6 (1.4 for UF1-PAN and 1.7 for UF10-PAN) showed that these membranes concentrated other substances with close or higher molecular weight cut-off together with polyphenols. In order to increase the concentration factor, it is necessary to conduct research with other membranes or to include other membrane processes. For example, Cassano et al., in 2011, used a combination of ultrafiltration and osmotic distillation in order to produce concentrated pomegranate juice [26]. Toker et al., in 2014 [30], established that polyphenolic concentration depended on the MWCO of the membrane during ultrafiltration of blood orange juice. In most cases, when the molecular weight cut-off of the membrane increases, the rejection decreases. For example, higher rejections of total polyphenols for 20 kDa membrane than for 30 kDa membrane during ultrafiltration of Valencia orange juice were published by Qaid et al. [24]. There are literature data that differ. Galanakis et al., in 2013 [31], found higher rejections of total polyphenols and anthocyanins during ultrafiltration of winery sludge with a 20 kDa membrane than with 1 kDa and 100 kDa. This could probably be explained by the formation of complexes between the polyphenols and some polymeric matrix compounds, e.g., proteins released during the extensive enzyme-catalyzed degradation of the rose petal byproduct. It was also proved that polyphenolic concentration during ultrafiltration of apple juice depends on the membrane material—hydrophilic or hydrophobic [26]. Wei et al., in 2007 [32], established an increase in the concentration factor and recovery of total polyphenols during the ultrafiltration of apple juice with a rise infeed concentration. According to Uyttebroek et al., the phenolic compounds from apple pomace can be successfully concentrated by nanofiltration [33], as well as in aqueous and ethanolic propolis extracts [34].

**Figure 5:**
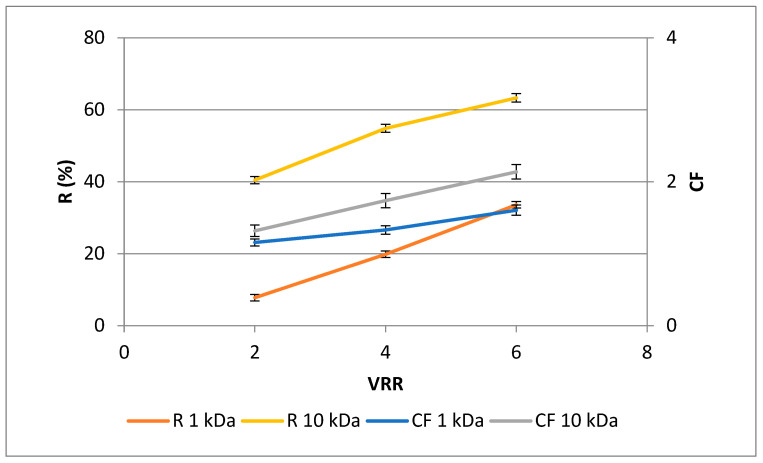
Dependences of concentration factor (CF) and rejection (R, %) of total solids (dry matter) in rose petal extracts on the VRR during ultrafiltration with 1 kDa (UF1-PAN) and 10 kDA (UF10-PAN) membranes.

Figure 5 presents the concentration factors and rejections of total solids depending on the VRR. Both parameters increased with the VRR rise from 2 to 6. Comparing the two membranes studied, it can be seen that the 10 kDa membrane had higher values of these two parameters (*p* < 0.05), except for the concentration factor at VRR 2. The maximal value of rejection (63.3%) was observed at VRR 6 when UF10-PAN was used for the concentration factor—2.13 at the same working conditions. This shows the possibility of ultrafiltration to concentrate dry matter. Similar investigations for other foods were published by Farnasova et al. [35] with potato juice. Cassano et al., in 2011, established a linear dependence between the total soluble solids and the duration of the process, as well as an exponential increase in the dynamic viscosity with an increase in total soluble solids during the concentration of pomegranate juice [26]. Acosta et al., in 2014 [25], established the highest rejection of total solids with a 5 kDa membrane and the lowest with 150 kDa membrane during ultrafiltration of blackberry (*Rubus adenotrichus* Schltdl.) juice. Within the investigated range of molecular weight cut-off, the authors found better rejection for 5 kDa than for the 1 kDa membrane.

**Figure 6:**
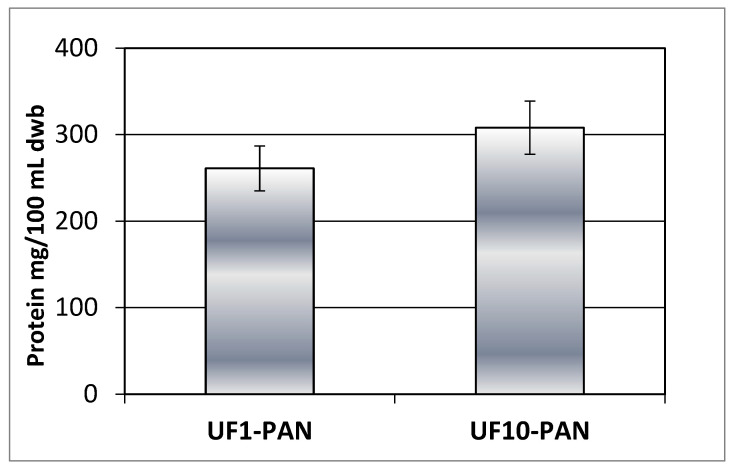
Protein content (mg BSAE/100 g dwb) of retentates (VRR 6) obtained during ultrafiltration with UF1-PAN and UF10-PAN.

The results in Figure 4 and Figure 5 imply that polyphenols could form complexes with some polymeric matrix compounds, e.g., proteins released during the extensive enzyme-catalyzed degradation of the rose petal byproduct, thus exhibiting similar concentration behavior during ultrafiltration with both the 1 kDa and 10 kDa membranes. This assumption is supported by the similar values obtained for the protein content of retentates at VRR 6 (Figure 6). Comparing the 50 kDa membranes produced from polyacrylonitrile (PAN), polyethersulfone (PES), and polyvinylidene fluoride (PVDF) during ultrafiltration of apple juice, Cai et al., in 2020 [27], established the highest protein content for the PAN membrane.

**Figure 7:**
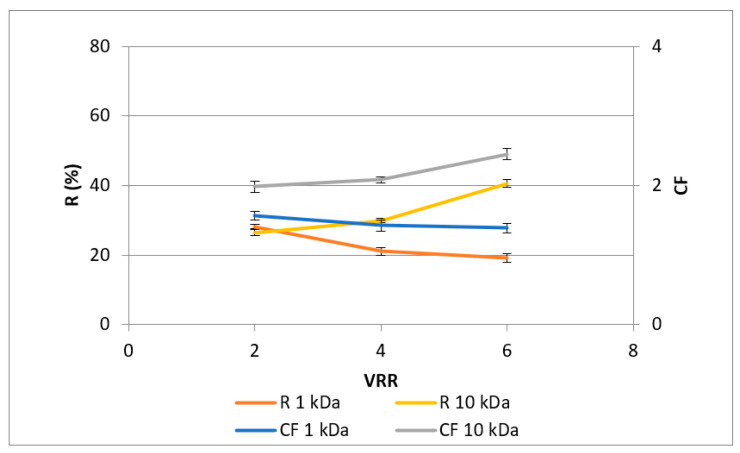
Dependences of concentration factor (CF) and rejection (R, %) of redox-active antioxidants (FRAP assay) in rose petal extracts on the VRR during ultrafiltration with 1 kDa (UF1-PAN) and 10 kDA (UF10-PAN) membranes.

**Figure 8:**
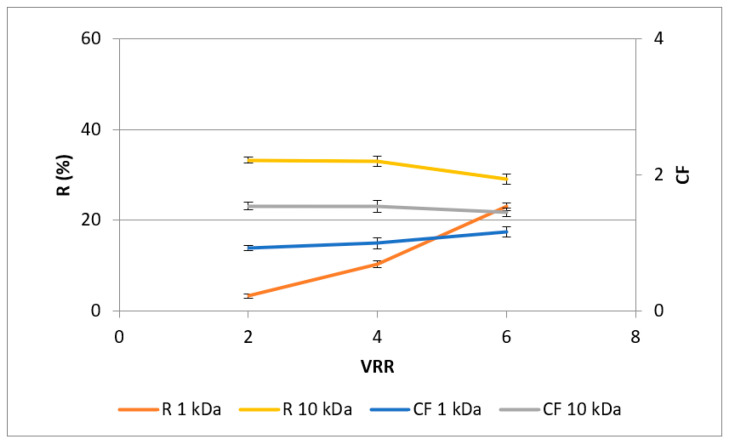
Dependences of concentration factor (CF) and rejection (R, %) of radical scavenging antioxidants (DPPH assay) in rose petal extracts on the VRR during ultrafiltration with 1 kDa (UF1-PAN) and 10 kDA (UF10-PAN) membranes.

The two assays employed in this study reflect different mechanisms for assessing antioxidant capability. The FRAP test analyzes the overall quantity of redox-active substances, whereas the DPPH assay evaluates the capacity of plant extracts to scavenge free radicals [36]. In general, the changes in the total antioxidant capacity (Figure 7 and Figure 8) correspond to the concentration behavior of total polyphenols. Comparing both membranes used, it could be seen that the highest values of rejection and concentration factor of redox-active antioxidants (FRAP assay) were observed at VRR 6 with the UF10-PAN membrane—40.5% and 2.5, respectively. Concerning the radical scavenging antioxidants (DPPH assay) presented in Figure 6, it can be concluded that the highest values of rejection and concentration factor were obtained again for VRR 6 and the UF10-PAN membrane—29.1% and 1.5, respectively. Some authors found that the rejection of anthocyanins decreased in logarithmic proportion when the MWCO increased in the ultrafiltration of roselle extract (*Hibiscus sabdariffa* L.) [12]. The total anthocyanins and total ellagitannins increased linearly with the MWCO decrease during the ultrafiltration of blackberry (*Rubus adenotrichus* Schltdl.) juice [23]. In contrast, Conidi et al., in 2017 [37], established a lower rejection value for total antioxidant activity with a 1 kDa membrane than with 2 and 4 kDa.

**Figure 9:**
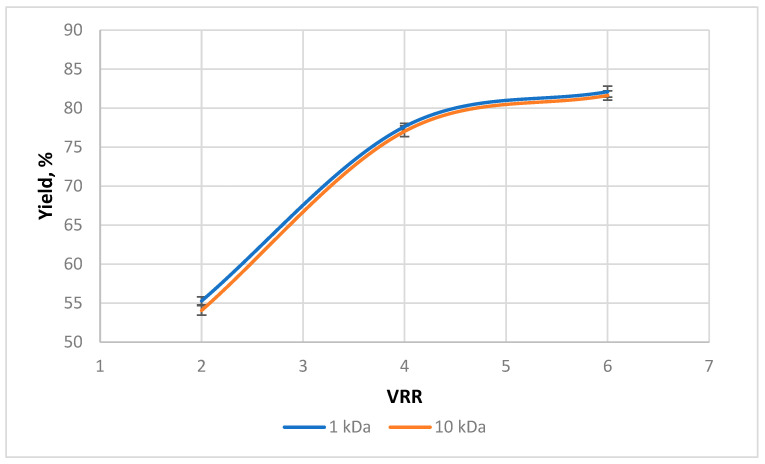
Effect of VRR on the polyphenols yield during ultrafiltration of rose petal extracts with 1 kDa (UF1-PAN) and 10 kDA (UF10-PAN) membranes.

The polyphenols yield during ultrafiltration at different VRRs is given in Figure 9. The increase in the concentration level led to a yield increase for both membranes. The results show a statistically insignificant difference between the two membranes at all VRRs (*p* > 0.05). This could probably be explained by the close values of polyphenolic content in permeates obtained with two membranes.

## 4. Conclusions

The results show that the permeate flux decreased with a rise in VRR and increased with a rise in transmembrane pressure and feed flow rate. At the beginning of the process, the highest flux was with the UF1-PAN membrane, but at the end of the process, it was with the UF10-PAN membrane. The total polyphenols of the retentates increased by 27–39% and 26–67% during ultrafiltration with the 1 kDa and 10 kDa membranes, respectively, with the highest value obtained for the 10 kDa membrane at VRR 6. The highest concentration factor and rejection of total solids, total polyphenols, redox-active antioxidants, and radical scavenging antioxidants were obtained at VRR 6 with the UF10-PAN membrane. The results obtained reveal the potential of ultrafiltration for the recovery of polyphenols from rose petal byproduct to increase the antioxidant capacity of retentates. This polyphenol fortification is also worthwhile from a technological point of view; it is an approach to quality improvement in food processing in general.

## Figures and Tables

**Table 1:** Contents of total polyphenols (mg GAE/100 mL) in rose petal extracts during ultrafiltration with different molecular weight cut-off membranes.

Extract Type	MWCO	
	1 kDa	10 kDa
Feed solution	246 ± 11 A	246 ± 11 A
Permeate	220 ± 10 aA	226 ± 10 aA
Retentate (VRR = 2)	332 ± 15 aB	310 ± 14 aB
Retentate (VRR = 4)	313 ± 14 aB	374 ± 17 bC
Retentate (VRR = 6)	341 ± 15 aB	410 ± 18 bC

Different lowercase letters within a row show significant differences (*p* < 0.05). Different capital letters within a column present significant differences (*p* < 0.05).

## Data Availability

Data are contained within the article.
